# Spatial Abilities for Architecture: Cross Sectional and Longitudinal Assessment With Novel and Existing Spatial Ability Tests

**DOI:** 10.3389/fpsyg.2020.609363

**Published:** 2021-02-02

**Authors:** Michal Berkowitz, Andri Gerber, Christian M. Thurn, Beatrix Emo, Christoph Hoelscher, Elsbeth Stern

**Affiliations:** ^1^Department of Humanities, Social and Political Sciences, ETH Zurich, Zurich, Switzerland; ^2^ZHAW School of Architecture, Design and Civil Engineering, Institute of Urban Landscape, Winterthur, Switzerland

**Keywords:** spatial abilities, architecture, higher education, gender, test performance

## Abstract

This study examined individual differences in spatial abilities of architecture students. Students at different educational levels were assessed on spatial ability tests that varied in their domain-specificity to architecture, with the hypothesis that larger differences between beginner and advanced students will emerge on more domain-specific tests. We also investigated gender differences in test performance and controlled for general reasoning ability across analyses. In a cross sectional study, master students (*N* = 91) outperformed beginners (*N* = 502) on two novel tests involving perspective taking and object composition, as well as on a standardized visualization of cross-sections test, but not on a standardized mental rotations test. Longitudinally (*N* = 117), spatial performance improved after the first bachelor year on visualization of cross-sections, object composition and mental rotation. Although both genders showed higher spatial test performance with increased experience, male students outperformed females across tests and levels of education. The results overall confirmed improvements in spatial performance during architecture studies, with partial support for the domain-specificity hypothesis. A gender gap among advanced students calls for further examining architecture-specific spatial thinking.

## Introduction

Architecture is famously described as “the thoughtful making of space” (Kahn, 1957). When architects design a building, for example, they engage in a multi-step and iterative process of manipulating spatial configurations, switching between perspectives, scales or forms of representation, and considering both aesthetic and functional requirements (Rowe, 1987; Akin, 2001; Cross, 2011). Although designing in architecture requires a multitude of skills, mentally visualizing spatial transformations is considered integral to it. Spatial thinking goes beyond architecture, as it has been shown to be a marker for success in several domains of science, technology, engineering and mathematics (STEM) (Wai et al., 2009; Uttal et al., 2013b). Psychological research on spatial thinking abilities has indeed been extensive in fields such as engineering, chemistry and mathematics (Sorby, 2007; Stieff et al., 2012; Newcombe et al., 2019). In the present research, we studied individual differences in spatial thinking abilities of architecture students at various points during their studies. Drawing on expertise research and findings on the benefits of spatial ability training (Uttal and Cohen, 2012; Uttal et al., 2013a), our basic assumption was that acquiring experience in architecture studies improves spatial abilities. Given different types of spatial ability, our main goal was to find out whether some forms of spatial thinking are likelier than others to be influenced by acquired experience in architecture. To do so, we combined standardized spatial ability tests with novel tests designed to be more specific to architectural tasks. Additionally, considering a male advantage in spatial ability tests performance (Levine et al., 2016), we investigated whether gender^1^ gaps exist among architecture students.

### Spatial Abilities and Their Measurement

Spatial thinking abilities generally refer to the mental processing and manipulation of spatial information such as shapes, locations, relations between objects or directions of movement (Newcombe and Shipley, 2015). Several typologies of spatial abilities have been proposed over decades of research on human cognitive abilities, with partially overlapping distinctions and no complete agreement (McGee, 1979; Linn and Petersen, 1985; Lohman, 1988; Carroll, 1993; for reviews see Hegarty and Waller, 2005; Uttal et al., 2013a). Nonetheless, most models acknowledge *spatial visualization* (SV) as the ability to perform complex and multi-step mental transformations of objects, often in three dimensions. Some researchers have included visualization of rotation under this factor (e.g., Carroll, 1993), while others identified mental rotation as distinct from SV (Linn and Petersen, 1985; Lohman, 1988). Here, we adapted the broader definition of SV, hence including mental rotation. Tests measuring SV are, for example, the Paper Folding Test (Ekstrom et al., 1976) and the Mental Rotations Test (Vandenberg and Kuse, 1978). *Spatial orientation* is another type of spatial ability identified in several models, though somewhat less consistently than SV (Uttal et al., 2013a). It refers to the ability to change one’s own viewing perspective of given objects, rather than performing transformations of object-parts. Comparing to SV tests, there are fewer tests of spatial orientation, some examples being The Spatial Orientation Test (Hegarty and Waller, 2004) and the Visualization of Views (Guay and McDaniels, 1976). Tasks that require changes in the imagined (or real) viewing point are also termed *egocentric*, whereas tasks in which the observing position is constant are *allocentric* and comprise the majority of SV tests (Kozhevnikov et al., 2013). A typology proposed by Uttal et al. (2013a) provides further dimensions for classifying spatial ability tasks. According to this model, the information in a given task can be either intrinsic—if focus is placed on features within a single object, or extrinsic—when the relations between different objects are targeted. Additionally, tasks are classified as either static—when no transformation to the objects is required, or dynamic—in case a transformation is involved (e.g., rotation, folding). Many SV tests are of the intrinsic-dynamic type, since a transformation is performed on a single object.

Many STEM subjects, including architecture, require the visualization and mental transformation of complex objects in three dimensional space, and therefore SV has been frequently studied in relation to STEM learning (Hambrick and Meinz, 2011; Vaci et al., 2019). Changes in perspective are also frequently required in several STEM domains, and are essential in the design process in architecture (e.g., Sutton and Williams, 2011). However, most spatial ability tests were not designed to assess skills that are special to one discipline or another. Rather, items on spatial ability tests are typically “de-contextualized” in order to minimize dependency on prior knowledge. Spatial skills measured this way are regarded particularly important for novices, whose domain-specific knowledge is low (Uttal and Cohen, 2012). It is known that with expertise, domain specific abilities are more likely than general abilities to develop and distinguish between experts and novices, although both domain-specific and domain-general abilities are relevant to performance (e.g., Hambrick and Meinz, 2011; Vaci et al., 2019). To some extent, this has been shown within the realm of spatial abilities, namely that spatial thinking becomes specialized with expertise in certain STEM domains (Hegarty et al., 2009; Stieff et al., 2012; Uttal and Cohen, 2012; Kozhevnikov et al., 2013; Shipley et al., 2013). For example, expert geologists outperformed chemists on a task simulating the process of inferring spatial properties of rock formation, but not on a standard mental rotation test (Shipley et al., 2013). Similarly, advanced dentistry students outperformed beginners on a novel, domain-specific test of tooth cross sectioning, but not on other, standardized spatial ability tests (Hegarty et al., 2009). In these studies, prior knowledge was not a precondition to solving the domain-specific task, but those who have acquired it had an advantage in performance comparing to novices. Yet, attempts to develop domain-specific measures of spatial abilities are few. Building on these studies and on expertise research more broadly, we assumed that expertise in architecture intensively trains some forms of spatial thinking more than others.

### Previous Research on Spatial Abilities in Architecture

Relatively few quantitative studies have focused specifically on spatial abilities of architecture students. The need for more research in this area has been pointed out by several authors within architecture (Sutton and Williams, 2011; Cho, 2012). More often, architecture students were studied together with engineering students, who all undergo spatially demanding courses such as technical drawing and descriptive geometry (Leopold et al., 2001; Williams et al., 2008). For example, Leopold et al. (2001) found improved performance on standardized spatial ability tests among beginner engineering and architecture students after taking introductory engineering graphics courses. The improvements were especially apparent when the courses involved sketching and ‘hands-on’ tasks. Williams et al. (2008) assessed spatial abilities of students from several engineering and creative design fields, including architecture. Based on tests suitable for a wide range of technical domains (Blasko et al., 2004; Sutton et al., 2007), engineering students showed an advantage on tasks more typical to engineering training. Conclusions regarding architecture student were, however, very limited in this study due their low proportion among all students. In another study, Sutton and Williams (2011) assessed only architecture students on the same test battery and found improved performance among beginner students, particularly after their first academic semester. The improvements were reported only on global scores, thus results for specific tests are not known. Moreover, no information was provided regarding sample size, statistical analyses or test properties in this study. Some researchers focused on tasks requiring domain-specific knowledge such as plan drawings, often with very small groups of students (Cho, 2012), while others relied on descriptive analyses for inferring specific difficulties in test performance (Nagy-Kondor and Sörös, 2012). While the above research overall confirmed the importance and malleability of spatial skills during architecture studies, large scale studies that specifically focus on architecture students and go beyond the bachelor level are currently not available. Moreover, assessments often relied on general spatial ability tests rather than on tasks that simulate design tasks in architecture. Finally, studies have usually not controlled for correlates of spatial abilities such as general reasoning ability, which could potentially account for group differences in spatial performance (Lohman, 1996).

### Gender Differences in Spatial Abilities

Among tests of human cognitive abilities, spatial ability tests are the only ones yielding substantial gender differences favoring males, especially in tasks involving mental rotation (Linn and Petersen, 1985; Neuburger et al., 2015; Levine et al., 2016; Xu et al., 2016). The assumed reasons for gender gaps in spatial abilities are manifold, and no consensus exists regarding the relative contribution of social and biological factors to these differences (Newcombe, 2007; Miller and Halpern, 2014; Tarampi et al., 2016). It is, however, widely acknowledged that spatial abilities are highly influenced by accumulated experience with spatial tasks and activities, and that these differ between males and females (Levine et al., 2016). Although research showed that spatial abilities are malleable (Uttal et al., 2013a), males and females tend to gain similarly from spatial ability training, resulting in persistent gaps in performance (Terlecki et al., 2008). Whether such performance differences explain at least partly the gender-gap in choosing careers in STEM-fields is discussed controversially (Halpern et al., 2007; Ceci et al., 2014). STEM students generally outperform non-STEM students on spatial ability tests (Peters et al., 2006; Wai et al., 2009), implying both self-selection of high-spatial ability students to STEM as well as improved spatial abilities as a result of higher education. However, gender differences on mental rotation tasks are found also among STEM students (Gorska et al., 1998; Peters et al., 2006; Sorby et al., 2013). Some of the studies cited above found such differences among architecture students as well, although there are inconsistencies and too few studies in this particular population (Leopold et al., 2001; Sutton and Williams, 2011; Nagy-Kondor and Sörös, 2012).

### The Present Research

To address some of the research gaps described above, we conducted a study in a large sample of architecture students, both at a beginner and an advanced level. We assessed spatial abilities with both existing, generalized spatial ability tests, and tests we developed as specifically relevant to architecture, while additionally controlling for general reasoning ability. While we assumed that both SV and spatial orientation are essential in architectural work, we hypothesized that existing tests may not capture some of the spatial mental processes that evolve in the course of specialization. As described above, one mental process that is crucial in architecture design is the ability to visualize changes in perspective. For example, architects frequently switch between viewing multiple objects such as buildings and streets from a bird’s eye view and from a user’s perspective. They also switch between different scales of objects and scenes (i.e., ‘zooming in and out’) and between two- and three-dimensional representations. As mentioned, relative to allocentric tests, fewer perspective-taking tests are available, particularly with complex objects. Our pilot testing (see section “Test Development and Preparatory Work”) additionally confirmed that existing tests of spatial orientation were too easy for architecture students. Thus, we aimed to design tests that would simulate the complexities of architecture tasks requiring changes in perspective. We developed two tests that are primarily egocentric. *The Urban Layout Test* (ULT) simulates switching between top- and ground views of an urban landscape, which additionally involves changes in scale and representation. The *Indoor Perspective Test* (IPT) simulates switching between internal and external viewpoints of a single structure. Assuming that changes in perspective qualify as a dynamic process, the ULT would be classified as *extrinsic-dynamic* according to the typology of Uttal et al. (2013a), while the IPT as *intrinsic-dynamic*. Another type of mental transformation relevant to architecture is the composing and decomposing of objects in space, or ‘filling in spaces’. During the design process, architects may begin from a given space and gradually transform and manipulate it by adding or subtracting volumes, by changing shapes and features or by rearranging their location. To our knowledge, available tests tapping this object-composition ability are mostly object assembly tasks with two-dimensional stimuli, such as the Revised Minnesota Paper Form Board Test (Likert and Quasha, 1970). In the architectural design process, such puzzle-like work needs to be done with more complex, three-dimensional objects. Thus, the third test, *Packing*, is essentially a 3D object-assembly task designed to simulate the combining and fitting together elements of design. It is primarily an allocentric task, and, in order to place the focus only on mental composition, we specifically excluded mental rotation as a means for solving this test. This task may also be classified as *extrinsic-dynamic* according to Uttal et al. (2013a).

Similar to previous work with geology and dentistry students (Hegarty et al., 2009; Shipley et al., 2013), the tests were designed to be solvable without prior knowledge in architecture. At the same time, the tests were meant to simulate architectural tasks both at the level of the mental process (i.e., activating spatial thinking common in architectural training and work), as well as by using stimuli that bare similarity to actual architectural objects, albeit in more abstract form. Designing the test items strongly relied on a collaboration with expert architects in our team, and was inspired by typical tasks given to architecture students. Thus, students who have gained more knowledge and experience in architecture were expected to have an advantage in performing these tasks. Additionally to the new tests, we included two widely used spatial ability tests that were previously linked with STEM achievements: the Mental Rotations Test (MRT; Peters et al., 1995) and the Mental Cutting Test (MCT; CEEB, 1939). Both tests are regarded measures of spatial abilities relevant to a wide variety of STEM domains. Among these, the MCT, which requires the visualization of cross-sections, has been frequently included in studies with engineering students (Tsutsumi et al., 2005; Sorby, 2009) and may capture more architecture-relevant ability than the MRT, since drawing cross-sections is an integral part in architectural work. Whereas we assumed that all these tests tapped spatial thinking that is important in architecture tasks, we expected that tests more specifically tailored to architectural work to better distinguish between levels of expertise. Finally, to assess general reasoning we included a figural-matrices test from a standard intelligence test (Amthauer et al., 2001), which required inductive reasoning with non-verbal stimuli.

We conducted two studies: In Study 1, we compared test performance between beginner and advanced architecture students on the new tests and on two standard spatial ability tests. In Study 2, we retested a sub-sample of participants from Study 1 one year later in order to track changes in performance. In line with the rationale described above, we expected advanced architecture students to outperform beginners on all spatial ability tests, but hypothesized domain-specificity of these differences, so that tests more closely simulating mental processes that are central in architecture studies will yield larger beginner-advanced differences. Similarly, we expected that in the course of architecture studies, students will improve on all spatial ability tests, with gains being more pronounced on those capturing spatial mental processes more specific to architecture. Given previous findings on gender differences in spatial abilities, we assumed gender differences favoring males may emerge among architecture students on some spatial ability tests, especially when mental rotation is involved. Nonetheless, since we focused on students who selected a spatially demanding area of study and included novel tests not confined to mental rotation, we could not make a strong hypothesis regarding the magnitude of gender differences. Similarly, we expected both genders to improve their spatial skills after gaining experience in architecture, and explored whether initial gender differences in spatial performance decreased.

## Study 1

In this cross-sectional study, we compared the performance of beginner and advanced architecture students from different schools of architecture on the three new spatial ability tests, two existing tests and one standardized test of general reasoning ability, which served as a control measure.

### Method

#### Test Development and Preparatory Work

The tests were developed in an interdisciplinary team and included discussion rounds with experts in both architecture and psychology. Pretesting was conducted among experts and non-experts until a final selection of items was made (more details are described in Gerber and Berkowitz, 2020). Prior to developing the new tests, a pilot study was conducted in which several spatial ability tests were administered to architecture students from different schools and degrees (*N* = 186; Gerber et al., 2019). This study revealed that scores on known perspective taking tests (Object Perspective Taking Test, Hegarty and Waller, 2004; and Visualization of Views; Guay, 1977) approached ceiling, confirming a need for more challenging perspective taking tests. The MRT and the MCT showed sufficient difficulty and variation. Additionally, the MCT, but not the MRT, distinguished between beginner and advanced architecture students, presumably indicating that visualization of cross-sections is more intensively trained in architectural work then mental rotation as assessed by the MRT.

#### Sample

A total of 593 architecture students participated in the study (49.7% female; *M* = 21.25 years, *SD* = 2.82 years). Students either in their first semester at an undergraduate program (*N* = 502) or in a masters’ degree program (*N* = 91) were recruited from three higher education institutes in Switzerland. Two academic institutes were technical universities (*n*_1_ = 277, 50% female; *n*_2_ = 213, 56% female) and one was a university for applied sciences (*n*_3_ = 103, 36% female). No admission exams are conducted in any of the institutes. Admission to the technical universities requires a high-school diploma of the higher track of secondary school (‘Gymnasium’), whereas the applied university enables admission also from vocational secondary school tracks. The core of architecture studies in all institutes is the design studio, which is highly similar across institutes. The main difference between institutes’ curricula is that the technical universities put more emphasis on theory and historical context, whereas the applied university is more practically oriented. There were no significant differences in gender distributions between degrees across institutes [Mantel–Haenszel chi-square with continuity correction χ_*MH*_^2^ (1) = 0.45, *p* = 0.50]. However, adjusted for degree, gender distributions across institutes significantly differed [generalized Cochran–Mantel–Haenszel statistic *M*^2^ (2) = 10.25, *p* = 0.01], with a lower proportion of female students in the applied university comparing to the other institutes. It should be noted that these gender distributions were roughly representative of the student population in each academic institute, thus were not likely due to self-selection to our study. Recruitment was done with the collaboration of architecture faculty via emails and class announcements. Participation was voluntary. Students were reimbursed with a 20CHF shopping voucher.

#### Measures

##### Spatial ability tests

###### Urban Layout Test (ULT)

**FIGURE 1:**
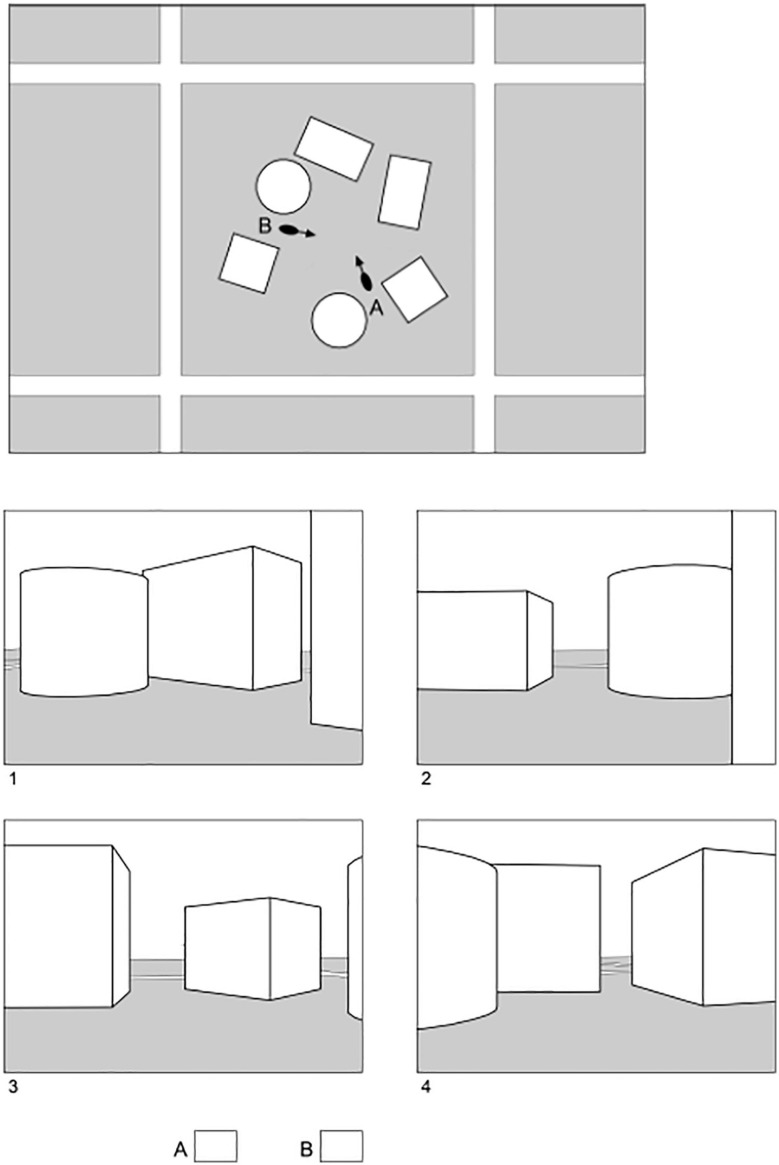
Example of an item on the Urban Layout Test.

This test assessed the ability to change one’s perspective with respect to an array of objects. It was designed to simulate the switching architects routinely make between viewing multiple objects such as buildings and streets from a bird’s eye view and viewing the objects from a user’s perspective. An example is presented in Figure 1. As shown, a top-view of an objects array was presented. Arrows were marked in two different locations on this image. The task was to imagine how the objects would look like from each of these standpoints. Thus, participants had to imagine themselves standing at each point and looking in the direction of the arrow. For each point, one of the four answer choices was correct.

The test consisted of eight questions with two answers each. The number of objects in an array was either six or eight (our pilot study showed that items with less objects were too easy to solve). Objects in the top view were displayed either in plan view (2D) or in axonometric view (3D). The distractors displayed views that were correct from other standpoints in the array. One point was given for each correct answer, so that on each question a maximum of two points was possible. The maximal score on this test was initially 16, though one item had to be excluded *post hoc* due to a drawing mistake, resulting in a maximal score of 15. Students were given 12 min to solve this test, based on pretests that showed this was sufficiently long for most students to work on all items.

###### Indoor Perspective Test (IPT)

**FIGURE 2:**
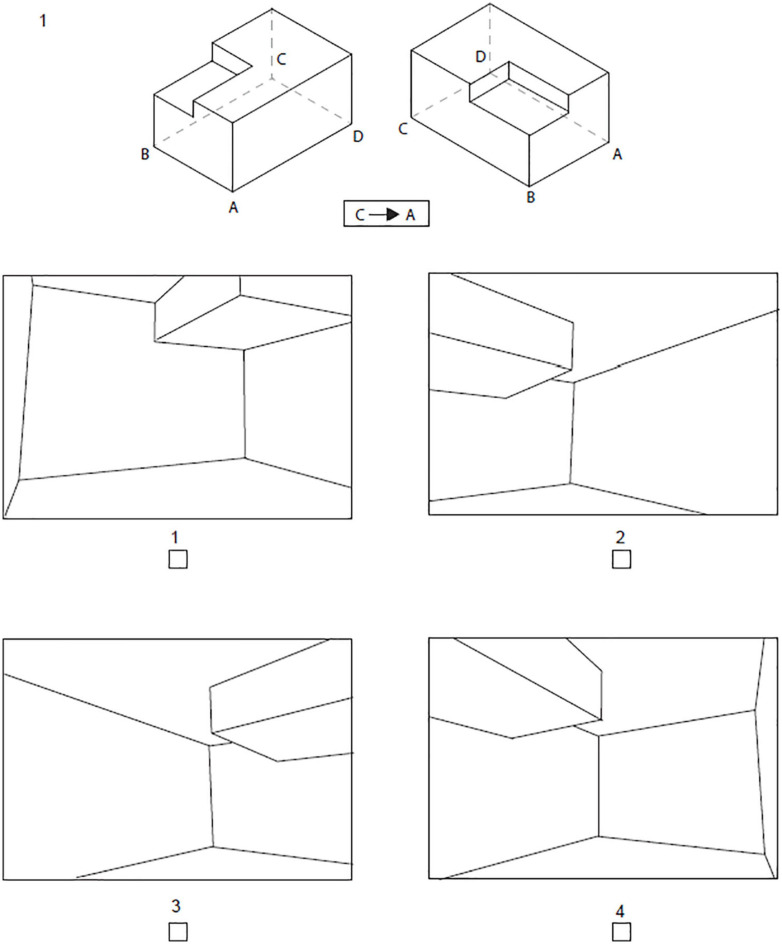
Example item from Indoor Perspective Test. Participants had to imagine themselves standing inside the object at point C and looking toward point A.

This test assessed the ability to visualize an object from ‘within’. Similar to the ULT, it intended to simulate changes in perspective, in this case between viewing an object externally and viewing its interior. An example is presented in Figure 2. At the top, an object was first shown from two external points of view. Four letters were marked at different points on the object. Participants were instructed to imagine themselves standing inside the object at one of those points and to look toward one of the other points. The exact points and direction of perspective were indicated below the objects by two letters and a one-way arrow between them, as shown in Figure 2. Only one of the four answers correctly displayed the view from the indicated point.

The test consisted of eight questions. Distractor answers displayed views that were possible from other points within the object. One point was given for each correct answer, thus the maximal score was 8 points. Students were given 6 min to solve this test, based on pretests that showed this was sufficiently long for most students to work on all items.

###### Packing

**FIGURE 3:**
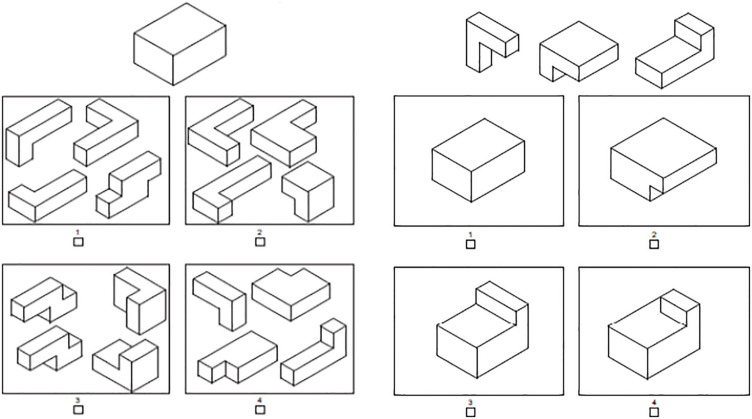
Example items from Packing. **Left:** whole-to-part item. **Right:** part-to-whole item.

This test assessed the ability to compose and decompose complex 3D objects. It intended to simulate the iterative process of object-composition in architectural design. The test included two types of items. One required selecting a set of elements that matched a given whole object. Participants were shown a target shape and four sets of smaller shapes (Figure 3, left). Only one of the four sets contained elements that could be put together to fit the target shape perfectly. The second type of items required selecting a whole object that matched a given set of elements (Figure 3, right). Here, participants saw a target set of shapes and four whole shapes. Only one of the whole shapes could result from combining the elements in the target set. Both types of problems were designed such that solutions could be reached only by mentally ‘moving’ the shapes vertically or horizontally, but not by mental rotation. Participants were thus explicitly required to imagine only vertical and horizontal movements and not to use mental rotation.

The test consisted of eight questions, four of which were ‘whole-to-parts’ and four ‘parts-to-whole’. Whole shapes were either cuboids or cylinders. The number of elements in each set of small shapes was either three or four. One point was given for each correct answer, thus the maximal score was eight points. Participants were given 8 min to solve this test, based on pretests that showed this was sufficiently long for most students to work on all items.

###### Mental Rotations Test (Vandenberg and Kuse, 1978; Peters et al., 1995)

In this test, participants were shown a drawing of a cubical figure and had to decide which two figures out of four were rotated versions of the target. There were 24 questions divided into two sets of 12 questions. In line with the procedure described by Peters et al. (1995), participants were given 3 min to complete each part. An answer was scored as correct only if both rotated figures were identified. Scores were the total of correct answers, ranging from 0 to 24.

###### Mental Cutting Test (MCT; CEEB, 1939)

In this test, participants were shown a drawing of a 3D shape being cut by a plain. Their task was to decide which of five alternatives was the resulting 2D cross-section. The original test consists of 25 items and takes 20 min. We used a shortened version with 10 questions and 8 min. Scores were the total of correct answers, ranging from 0 to 10.

##### General reasoning ability

The subscale *Matrices* from the well-established *Intelligence Structure Test 2000 Revised* (*IST*, Amthauer et al., 2001) served for assessing general reasoning ability. This test measures inductive reasoning with non-verbal stimuli, one of the indicators of fluid intelligence. On each item, participants were shown four drawings of two-dimensional shapes and were asked to select the next shape in the series out of five options. The test consisted of 20 items and participants were given 10 min to solve. One point was given for each correct answer, with a maximal score of 20.

#### Procedure

The study was approved by the ethics committee at ETH Zurich. All students signed an informed consent for participating in the study. Testing was conducted in groups in students’ respective institutions. All test materials were in paper-and-pencil form. Each test began with written instructions and examples. A trained experimenter provided general oral instructions before testing began. Administration of the MRT, MCT, and IST-matrices was done according to the standard procedure reported for these tests. Students worked individually. The order of tests was the same for all participants. The total testing time was 1 h and 15 min and included a break.

### Results

Table 1 presents descriptive statistics and reliability estimates of all measures. Conventional scale reliability estimates (i.e., Cronbach’s alpha based on Pearson correlations) for the new tests were below the recommended cut-off value (which is for Cronbach’s alpha: >0.70; Nunnally and Bernstein, 1994), but sufficient when the tests were combined to form one scale (31 items, α = 0.71). As argued by many researchers (Sijtsma, 2009; Dunn et al., 2014; McNeish, 2018; Savalei and Reise, 2019), the alpha estimate may be highly restrictive due to strong assumptions of unidimensionality and tau-equivalence (i.e., all items having the same unstandardized factor loading on the latent construct). Coefficient omega (Omega total, Dunn et al., 2014) is one alternative to alpha when strict unidimensionality and tau-equivalence cannot be assumed. With binary data, estimates based on tetrachoric correlations are suitable (Gadermann et al., 2012). These values appear in Table 1 under *ordinal omega total*. Additionally, Rasch models fitted the newly developed tests well, with all item fits being between the criteria of 0.75 < infit MSQ/outfit MSQ < 1.33 (Wright et al., 1994). The infit and outfit values are given in Appendix Table A1. Although heterogeneity across items existed in the new tests, these analyses overall indicate sufficient test-reliability.

**TABLE 1:** Descriptive statistics and reliability estimates of the study’s tests.

	**Raw Mean (*SD*)**	**Mean proportion correct (*SD*)**	**Skew**	**Min**	**Max**	**N Items**	**Cronbach’s α (95% CI)**	**Ordinal Omega total (95% CI)**
ULT	10.48 (2.50)	0.70 (0.17)	−0.45	2	15	15	0.57 (0.52–0.62)	0.72 (0.68–0.75)
IPT	5.71 (1.58)	0.71 (0.20)	−0.60	0	8	8	0.44 (0.37–0.51)	0.64 (0.60–0.69)
Packing	5.40 (1.70)	0.67 (0.21)	−0.30	0	8	8	0.53 (0.47–0.58)	0.69 (0.66–0.73)
MRT	13.40 (4.92)	0.56 (0.20)	−0.04	0	24	24	0.86 (0.85–0.88)	0.93 (0.92–0.94)
MCT	6.35 (2.37)	0.63 (0.24)	−0.30	0	10	10	0.70 (0.67–0.74)	0.84 (0.82–0.86)
IST-M	11.77 (2.81)	0.59 (0.14)	−0.16	3	19	20	0.63 (0.59–0.68)	0.79 (0.76–0.81)

*ULT, Urban Layout Test; IPT, Indoor Perspective Test; MRT, Mental Rotations Test; MCT, Mental Cutting Test; IST-M, Intelligence Structure Test – Matrices.*

#### Construct Validity

The correlations between tests are shown in Table 2. The five spatial ability tests were positively and moderately correlated, indicating a partial overlap in the measured abilities. Thus, each test has captured some unique skills, whereas other elements were common across the tests. A confirmatory factor analysis (CFA) on the five spatial ability tests indicated that one-factor best fitted the data, thus confirming their validity as tests of spatial ability (χ^2^ = 6.60, df = 5, *p* = 0.25; RMSEA = 0.02, 90% CI = 0.00–0.07; CFI = 0.997; SRMR = 0.02). The weaker correlations between the spatial tests and the reasoning test further confirmed this construct validity. Models with separate factors for allocentric (MRT, MCT, Packing) and egocentric tasks (ULT, Indoor), or for the three novel tests and the two existing tests resulted in poorer fit and high overlap between factors. Thus, non-overlapping elements were presumably task-specific.

**TABLE 2:** Correlations between observed test scores.

	**1**	**2**	**3**	**4**	**5**
(1) ULT	−				
(2) Packing	**0.36**	–			
(3) IPT	**0.38**	**0.30**	–		
(4) MCT	**0.40**	**0.40**	**0.35**	−	
(5) MRT	**0.43**	**0.42**	**0.37**	**0.39**	−
(6) IST-M	**0.21**	**0.16**	**0.12**	0.05	**0.26**

*All bolded values are significant at *p* < 0.01. ULT, Urban Layout Test; IPT, Indoor Perspective Test; MRT, Mental Rotations Test; MCT, Mental Cutting Test; IST-M, Intelligence Structure Test – Matrices.*

#### Measurement Invariance

When investigating group differences, it is important to establish measurement invariance in order to ensure that the scales function similarly across groups (Van de Schoot et al., 2012). Although increasingly more studies examine the effects of item properties on differences in spatial tests performance (Bors and Vigneau, 2011; Boone and Hegarty, 2017), few formally test for measurement invariance (Xu et al., 2016). To check whether some test items showed differential item functioning (DIF) between groups (educational level, gender), we calculated for each item the Mantel–Haenszel chi-square statistic (Mantel and Haenszel, 1959). A corresponding effect size is available for this measure (ETS delta scale), with values smaller than 1 classified as negligible, values between 1 and 1.5 classified as moderate and values larger than 1.5 classified as large differences (Holland and Thayer, 1985). This analysis showed that between education levels (i.e., bachelor and master), none of the items on *Packing*, IPT, MCT, and MRT showed significant DIF values. On the ULT, only one item had a significant DIF (ΔMH = 1.86). Thus, for the most part, test items did not significantly differ in their functioning between groups, therefore comparing mean test scores across these groups can be assumed valid. Similar results were obtained for gender: No significant DIFs were found for *Packing*, MCT and MRT, whereas one ULT item and one IPT item showed significant DIFs (ΔMH = −1.99 and ΔMH = −1.56 respectively). We conclude that most items worked similarly for both genders.

#### Group Differences

##### Beginner and advanced students

To compare test performance between beginner and advanced students and between genders, we conducted a 2 (degree) × 2 (gender) MANOVA, first with scores on the five spatial ability tests and on the general reasoning test as dependent variables^2^. This analysis revealed a significant main effect for degree, *F*(6,581) = 7.16, *p* < 0.001, η_*p*_^2^ = 0.07 and a significant main effect for gender, *F*(6,581) = 12.71, *p* < 0.001, η_*p*_^2^ = 0.12. The degree-by-gender interaction was not significant [*F*(6,581) = 1.74, *p* = 0.11]. Next, we conducted the same analysis with general reasoning scores as a covariate. Although reasoning ability positively affected spatial ability scores across groups [*F*(5,581) = 15.07, *p* < 0.001, η_*p*_^2^ = 0.11], controlling for it yielded the same pattern of results: A significant main effect for degree [*F*(5,581) = 8.5, *p* < 0.001, η_*p*_^2^ = 0.07]; a significant main effect for gender [*F*(5,581) = 13.76, *p* < 0.001, η_*p*_^2^ = 0.11]; and no significant interaction effects. Thus, the pattern of results on the spatial ability tests could not be accounted for by differences in general reasoning ability.

**FIGURE 4:**
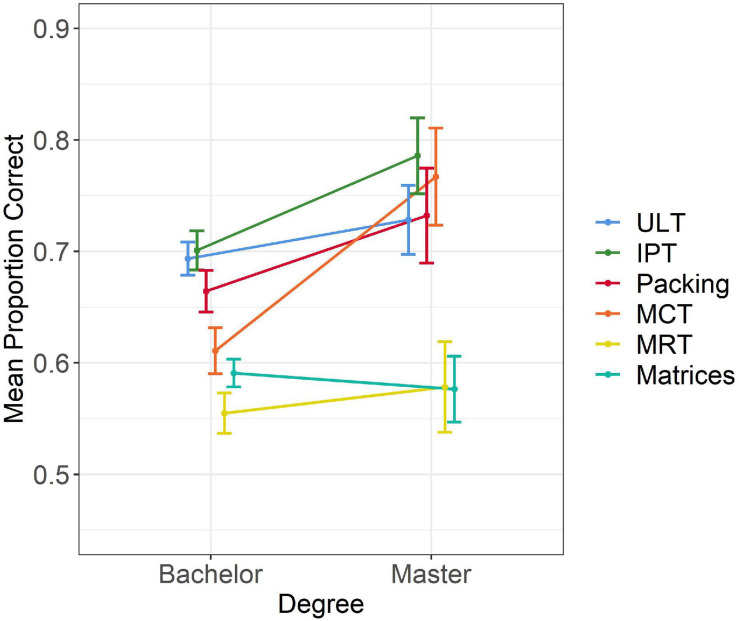
Performance on the six tests split by degree. Error bars indicate 95% confidence interval.

**FIGURE 5:**
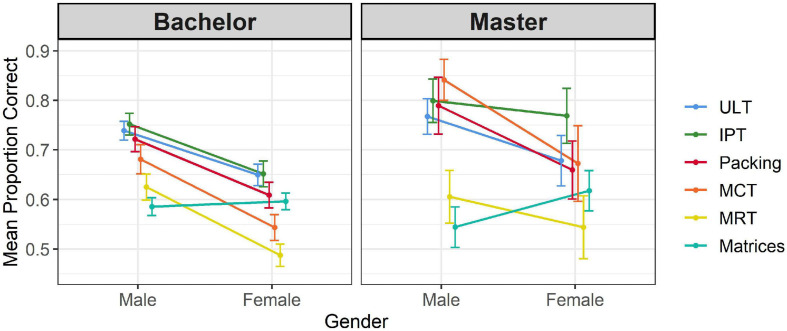
Test performance by gender and degree. Error bars indicate 95% confidence intervals.

The results for individual tests by degree and gender are displayed in Tables 3, 4 and Figures 4, 5 respectively. Along with η_*p*_^2^ values, we report Hedges’ *g* values of effect size, which is appropriate when group size differs substantially as in the case of the bachelor-master comparisons. For consistency, we calculated Hedges’ *g* in all analyses, which in cases of equal sample size is identical to Cohens’ *d* values. As shown, advanced students significantly outperformed beginners on *Packing*, IPT, and MCT, the last two showing stronger effects than the first. Differences on ULT, MRT, and *Matrices* did not reach significance. Male students outperformed female students on all spatial ability tests, whereas a small effect favoring females emerged on the general reasoning test. Although gender-by-degree interactions did not reach statistical significance, gender differences on MRT and IPT were slightly smaller among advanced students (among bachelor students, Hedges’ *g* = 0.53 and *g* = 0.70 respectively, among master students Hedges’ *g* = 0.49, and *g* = 0.65).

**TABLE 3:** Mean values and MANOVA results comparing test performance of beginner and advanced students.

	**Mean (*SD*) bachelor (*n* = 502)**	**Mean (*SD*) master (*n* = 91)**	***F***	***p***	**η_*p*_^2^**	**Hedges’ *g***
ULT	0.69 (0.17)	0.73 (0.15)	2.42	0.12	0.004	0.21
IPT	0.70 (0.20)	0.79 (0.16)	9.04	0.003	0.02	0.43
Packing	0.66 (0.21)	0.73 (0.20)	6.37	0.01	0.01	0.32
MCT	0.61 (0.23)	0.77 (0.21)	33.15	<0.001	0.05	0.68
MRT	0.55 (0.21)	0.58 (0.19)	0.68	0.41	0.001	0.11
IST-M	0.59 (0.14)	0.58 (0.14)	0.38	0.54	<0.001	–0.10

*ULT, Urban Layout Test; IPT, Indoor Perspective Test; MRT, Mental Rotations Test; MCT, Mental Cutting Test; IST-M, Intelligence Structure Test – Matrices.*

**TABLE 4:** MANOVA results comparing test performance between genders (across degrees).

	**Mean (*SD*) Males (*n* = 296)**	**Mean (*SD*) Females (*n* = 294)**	***F***	***p***	**η_*p*_^2^**	**Hedges’ *g***
ULT	0.74 (0.15)	0.65 (0.17)	23.38	<0.001	0.04	0.56
IPT	0.76 (0.17)	0.67 (0.21)	9.04	<0.01	0.02	0.48
Packing	0.73 (0.20)	0.62 (0.21)	26.91	<0.001	0.04	0.57
MCT	0.71 (0.23)	0.56 (0.22)	37.06	<0.001	0.06	0.65
MRT	0.62 (0.20)	0.50 (0.19)	19.86	<0.001	0.03	0.65
IST-M	0.58 (0.14)	0.60 (0.14)	6.84	0.01	0.01	–0.15

*ULT, Urban Layout Test; IPT, Indoor Perspective Test; MRT, Mental Rotations Test; MCT, Mental Cutting Test; IST-M, Intelligence Structure Test – Matrices.*

### Interim Discussion

The results of Study 1 revealed that advanced architecture students outperformed beginners on two of the novel spatial ability tests (IPT and *Packing*) as well as on the MCT, whereas differences on the ULT and MRT were smaller and non-significant. Reasoning ability was highly similar between bachelor and master students, and could not account for the differences in spatial performance. These results overall support the hypothesis that higher spatial ability goes along with higher expertise in architecture, and partially support our domain-specificity hypothesis. Specifically, since higher scores among advanced students emerged only on some of the tests, these tests potentially captured more architecture-specific skills than the other tests. At the same time, one of our novel perspective taking tests (ULT) did not yield the expected beginner-advanced differences. Because this test resembles tasks that are very common in architecture studies, we assume that performance on this test might have been sufficiently high at baseline (i.e., as a beginner), as discussed later. The MCT yielded the strongest difference, presumably indicating that visualization of cross-sections is central in architecture, as also emerged in our pilot study. Thus, the MCT may be more domain-specific to architecture than the MRT.

In addition to beginner-advanced differences, we found gender differences favoring males across the spatial tests, both among beginners and advanced students. Although some of these differences seemed smaller in the advanced group, these interaction effects were not statistically significant. Moreover, although general reasoning ability was positively correlated with spatial test performance, it could not account for the gender differences. These results are overall in line with previous findings on gender differences in spatial ability tasks (Levine et al., 2016). Our results extend these findings by revealing persistent gender differences also among students in a spatially demanding field as architecture, beyond the beginner level. Moreover, unlike in many other STEM fields, the almost even male-to-female ratio in architecture programs, reflected in our sample as well, enabled a solid comparison of test performance. The effect size of the differences is, on one hand, smaller than those typically found in less selective populations (e.g., for MRT, values higher than 0.70 are common; Linn and Petersen, 1985; Voyer et al., 1995). Nonetheless, it is in the range reported for STEM students (e.g., 0.48 in Peters et al., 1995).

## Study 2

Study 1 focused on beginner-advanced comparisons based on cross-sectional data. In Study 2, we assessed spatial performance within one sample across time. As before, we hypothesized that spatial abilities will improve in the course of architecture studies, and we were interested to find out whether some types of spatial skills were more likely to change than others. To answer this question, we conducted a follow-up study in a subsample of beginner students who participated in Study 1. These students were given the five spatial ability tests about one year after the first measurement. We hypothesized that performance would improve to a greater extent on tests capturing skills that are frequently trained during architecture studies, than on tests demanding skills less central in these studies. As explained below, we administered modified versions of the three new spatial tests, but based the analysis on items that were included at both measurement points.

### Method

#### Sample and Procedure

Students in this sample were from the same higher education institutes that participated in Study 1. In Study 1, students were informed that follow up studies are planned, and gave their consent to be contacted again. About one year later, all participants received a personal email invitation to participate in a follow up study. A total of 117 bachelor students participated in this study (48 males, 69 females; 55, 20, and 42 from the three schools respectively). In order to test for self-selection effects to Study 2, we compared test scores at time 1 between these participants and the rest of the bachelor students who did not participate in Study 2. Among male students, no significant differences emerged on any of the tests, [*F*(6,238) = 0.32, *p* = 0.93]. Among female students, those who participated in Study 2 outperformed those who did not on the ULT [*F*(1,252) = 22.83, *p* < 0.001, η_*p*_^2^ = 0.08], MCT [*F*(1,252) = 10.08, *p* = 0.002, η_*p*_^2^ = 0.04], and reasoning [*F*(1,252) = 11.13, *p* = 0.001, η_*p*_^2^ = 0.04]. Thus, at baseline, female students who chose to participate in Study 2 had an advantage on two spatial tests and on general reasoning comparing to female students who participated only in Study 1. This self-selection effect was not found for male students.

The second measurement took place between 12 and 18 months after the first study, when students were at their second bachelor year. As before, participants received 20CHF for their participation. Testing took place in groups outside lecture times.

#### Measures

The five spatial ability tests from Study 1 were included in this study (ULT, IPT, *Packing*, MCT, MRT). We administered modified and slightly longer versions of ULT, IPT, and *Packing* (10 questions per test), which included most of the original items. These versions yielded slightly improved psychometric properties, as shown in Appendix Table A2. The tests are available in full version at: https://osf.io/jf5mx/. We again checked the items for DIF using the Mantel–Haenszel statistic. No scale showed significant DIF values between genders. To enable consistency of measurement across time, the analysis of score change was based only on test items that appeared at both time points. The overlap for the ULT was six items, for IPT five items and for *Packing* seven items. Thus, in the analyses reported next, test scores were calculated for the overlapping parts.

### Results

**FIGURE 6:**
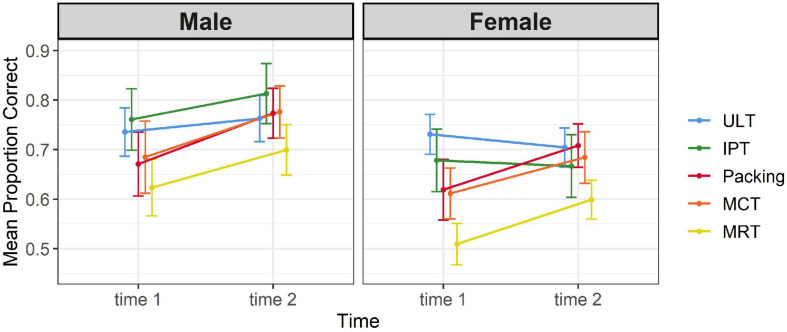
Test scores at two time points by gender. Error bars indicate 95% confidence intervals.

To examine whether test scores differed between the two measurement points, a mixed MANOVA was conducted with time as a within subject variable and gender as a between-subject variable. Scores on the five tests were the dependent variables. Scores on ULT, IPT, and *Packing* were based only on items that appeared in both measurement points, as presented in Table 5 (scores on the full versions appear in Appendix Table A3). There were significant effects of time [*F*(5,109) = 12.96, *p* < 0.001, η_*p*_^2^ = 0.37] and gender [*F*(5,109) = 3.60, *p* < 0.01, η_*p*_^2^ = 0.14], and a non-significant time-by-gender interaction [*F*(5,109) = 1.6, *p* = 0.56, η_*p*_^2^ = 0.04]. As shown in Table 5, there were significant improvements in mean test scores over time on *Packing*, MCT, and MRT, whereas scores on ULT and IPT were highly similar. These improvements were similar across genders, as shown in Figure 6. Male students outperformed females at both time points to a similar degree on all tests except on the ULT, on which gender differences were not significant [*F*(1,113) = 1.51, *p* = 0.22]. Similar results were obtained when controlling for reasoning ability and including academic institute in the analysis [time: *F*(5,107) = 10.92, *p* < 0.001, η_*p*_^2^ = 0.34]; gender: [*F*(5,106) = 4.55, *p* < 0.01, η_*p*_^2^ = 0.18; non-significant timeXgender interaction: *F*(5,106) = 0.61, *p* = 0.69, η_*p*_^2^ = 0.03].

**TABLE 5:** Mixed MANOVA on test scores across time (*N* = 117).

	**Mean (*SD*) Time 1**	**Mean (*SD*) Time 2**	***F***	***p***	**η_*p*_^2^**	**Hedges’ *g***
ULT	0.73 (0.16)	0.73 (0.16)	0.00	0.88	0.00	−0.01
Packing	0.64 (0.24)	0.73 (0.18)	14.00	<0.001	0.11	0.44
IPT	0.71 (0.24)	0.72 (0.25)	0.60	0.44	0.01	0.06
MCT	0.64 (0.23)	0.72 (0.21)	23.22	<0.001	0.17	0.36
MRT	0.56 (0.19)	0.64 (0.17)	35.77	<0.001	0.24	0.47

## General Discussion

Previous research showed that spatial performance improves with training and expertise in spatially demanding domains (Uttal et al., 2013a). It is additionally known that domain-specific skills, including spatial abilities, develop with expertise (Hegarty et al., 2009; Shipley et al., 2013). In this study, three novel tests were introduced, which aimed to capture spatial mental processes frequently required in architecture studies. Our main hypothesis was that with accumulated experience in architecture studies, performance on a range of spatial ability tests would improve, with larger beginner-advanced differences expected in tests involving more architecture-relevant spatial abilities. Additionally, we were interested in potential gender differences in this population, given known advantages of males on some forms of spatial performance (Levine et al., 2016). Our data overall confirmed higher spatial ability among advanced architecture students, both cross-sectionally and longitudinally. To this end, the study extends previous findings and adds to a relatively limited body of research on spatial abilities in architecture. At the same time, the pattern of results was not entirely consistent across measures, thereby partially supporting our domain-specificity hypothesis. In the first study, two of the new tests (*Packing* and IPT) distinguished between architecture students at the beginning of their bachelor studies and students at the master level. Thus, these tests may have been sensitive to spatial abilities that develop in the course of architecture studies, as intended. Surprisingly, the ULT showed a much weaker and non-significant effect, although the type of perspective taking required in this test is very common to tasks in architecture studies. Of the standardized tests, the MCT showed an even stronger effect favoring master students, whereas the effect of MRT was small and not significant. The MCT has been previously used in research among engineering students and related disciplines, in which visualization of cross-sections is very common (Tsutsumi et al., 2005; Sorby, 2009). Thus, the MCT might be more influenced by experience with architecture tasks than the MRT. Importantly, the results could not be explained by differences in general reasoning ability, which did not significantly differ between the groups. In Study 2, we found that after one year of architecture studies, performance improved on *Packing*, MCT, and MRT. The findings for the first two tests were consistent with those of Study 1, whereas the MRT result was not. Furthermore, the improvement was greater on MCT and MRT than on *Packing*, contrary to our hypothesis. Surprisingly, performance on the two perspective taking tests was highly similar across time. We next discuss possible explanations for these results.

### Different Levels of Specialization

One possible explanation for the different results between studies might be that different advanced groups were considered. In Study 1, the advanced group consisted of graduate students during their master’s degree program, whereas students in Study 2 were bachelor students in their second year. Possibly, improved performance on some tests becomes more pronounced at an advanced level that is beyond the first academic year. For example, the higher performance of master students on the IPT but the absence of a significant score change after the first bachelor year could indicate that this type of perspective taking is more intensively trained in advanced years, or that more time is needed for improvements to emerge. In contrast, improvement on *Packing*, MCT, and MRT emerged early, indicating that these tests are highly sensitive to the spatial experience that is gained during the first stages of specialization. Moreover, the MCT and *Packing* distinguished between beginner and advanced students both at the master level (Study 1) and after one bachelor year (Study 2). Thus, both visualization of cross-sections, which is the focus of the MCT, and mental composition of objects, which is the focus of *Packing*, may continue to be demanding and malleable during advanced levels. In contrast, mental rotation, as assessed by the standard MRT, significantly improved after one bachelor year but did not significantly differ between master and bachelor students in the first study. Possibly, the ability tapped by this test is more malleable at the novice phase, although longitudinal data (i.e., into advanced degrees) would be needed in order to test this hypothesis. Nonetheless, the finding is consistent with previous conclusions regarding spatial ability and STEM more broadly (Uttal and Cohen, 2012). As discussed next, the weaker effects on the perspective taking tests, and in particular their absence on the ULT, are puzzling, because changes in perspective are intensively involved in architectural studies.

### Test Properties

Other factors that might have played a role in yielding the current results are related to test properties. First, there may have been differences in test difficulty. *Packing*, MCT, and MRT tended to be more difficult, on average, than the two perspective taking tests, as indicated by mean test scores. With a lower score to start from, improvements may have been more likely on these tests. Although we aimed to create difficult perspective taking tasks, the students in this study were quite successful on these tests already as beginners. One possibility is therefore that students were sufficiently competent in perspective taking at baseline, or, alternatively, that performance on these tests is less easily improved than on the other tests. In fact, little is known about the effects of training and experience on spatial orientation (i.e., perspective taking), as most of the available research focused on other types of spatial abilities (Uttal et al., 2013a). Second, higher variability in test items in the new tests might have attenuated true differences between these and the standardized tests with respect to effects of experience. Although a single underlying factor best represented the data across tests, further distinctions might have been underestimated due to heterogeneity in the new tests. Third, the overlap between new and existing tests may nonetheless indicate that the new tests were less domain-specific than intended. Relatedly, the results indicate that the types of spatial abilities assessed by both the general and the specific tests play a role in architecture training, and that their differential effects might by more subtle than we had assumed. Still, it is possible that in order to detect domain-specificity at the construct level, as well as differential links with architecture expertise, even more realistic and contextualized architectural tasks would be needed. Finally, it is interesting to note that the correlation between the two perspective-taking tests was not higher than their correlation with the other tests. Although this may be partly due to high item-heterogeneity, it may also indicate a distinction between types of perspective taking, which possibly differ in their development across architecture training. In the ULT, the focus was on spatial relations between objects, whereas in the IPT it was on spatial relations within a single object^3^, a distinction that maps well onto the intrinsic-extrinsic dimension suggested by Uttal et al. (2013a). While we assumed both to be highly important in architecture, the second may be somewhat more influenced by specialization, as indicated by the higher scores of master students on this test. Additionally, the egocentric position might have been more strongly manipulated in the IPT, whereas in the ULT other cues and alternatives might have been available for shifting the egocentric view. Further research would be needed in order to both replicate this finding and to test whether such a distinction emerges with other perspective-taking tests.

### Gender Differences

Consistent with previous findings (Peters et al., 2006; Levine et al., 2016), our data revealed gender differences favoring males across spatial ability tests, samples and educational levels. The equal share of males and females in architecture programs allowed for a more balanced comparison than usually possible in many other STEM disciplines, to which substantially more males than females enroll. The differences could not be explained by general reasoning ability, which showed either no difference or a slight advantage for female students. In Study 1, males’ advantage tended to be smaller on some tests among advanced students comparing to beginners, suggesting that increased expertise may help reducing this gender gap. However, our data did not indicate a decrease, nor an increase, in the gender gap after one year of study: both genders improved to a similar extent on some of the tests. Thus, although females’ performance improved with time, it remained lower than their male peers’ performance. Moreover, given self-selection of female students with higher baseline scores to Study 2, we suspect that some of the gender gaps in Study 2 were underestimated. The results are consistent with previous findings on improved spatial performance following training that was similar across genders, thus preserving the gap at a higher level of performance (Terlecki et al., 2008).

There are several possible implications of this finding. First, it is possible that a decrease in the gender gap may occur only at more advanced stages, and takes long to emerge without a focused intervention. If accumulated experience from early age plays a role in spatial gender-gaps, it may not be surprising that reducing it takes long as well. Second, it is possible that such a gap emerges on test performance, but would not be found in highly specialized, real-world architecture tasks. That is, although our novel tests simulated mental processes involved in architectural tasks, they are still inevitably reduced and less contextualized comparing to the actual tasks in the design process. Furthermore, research in other fields found that experts develop highly specialized skills and problems solving strategies that reduce the mental visualization effort (Stieff et al., 2012). A more detailed analysis of expert-novices task performance would be needed in order to find out whether this holds for architects as well. Finally, it is possible that after initial improvements in spatial performance across genders, a sufficient ‘threshold’ is already reached, beyond which remaining differences no longer play a crucial role. Considering enrollment to advanced degrees as one criterion for success in a field (Wai et al., 2009), gender differences in spatial test performance among advanced students may be seen as not crucial to performance in architecture, because students with too low spatial skills would have not made it into advanced programs. Nonetheless, criteria for ‘good architecture’ beyond academic degree are not well defined empirically. Therefore, it remains open whether and at which points in the course of specialization gender gaps in spatial ability play a significant role in men’s and women’s future success as architects.

### Limitations

The new tests described here measure a few but not all possible aspects of spatial thinking in architecture. Further types of tasks can be relevant and explored in future research. With regard to test properties, the new tests might have been less restrictive in their features comparing to typical psychometric tests. For example, mental rotation items vary only by angle and orientation of the arms, yielding high stimulus similarity and likely minimizing variations in solving strategies between items. In the present case, items varied on multiple dimensions, which, on one hand, contributes to their ecological validity, while at the same time might have compromised other psychometric properties. One way to better understand how architects solve these tests would be to qualitatively analyze their solving strategies. Additionally, this study focused only on architecture students. To determine the domain-specificity of spatial mental processes, comparing performance between architecture students and students in other disciplines (e.g., chemistry), especially after gaining substantial experience in their fields, would be needed.

## Conclusion

The current study contributes to research on spatial abilities in architecture and more broadly. First, the study confirmed an often made but not as often tested assumption that spatial abilities improve during architecture studies. Our data shows that such improvement appears already at the beginning of the professional track and is not unitary across measures. To further understand architecture-specific spatial thinking, future research needs to focus on a more detailed process analysis of test performance among experts and novices. Second, since spatial skills are highly sensitive to training, placing a more direct focus on these skills within the curriculum of beginner architecture students may be beneficial to both males and females. Although students already ‘train’ their spatial skills in tasks that are inherent to their courses, a focused training of specific skills may help beginner students, particularly those with initially poor spatial skills, to obtain a necessary level earlier, as has been shown with engineering students (Sorby, 2009). Regarding gender, our data raises two different concerns. On one hand, a consistent disadvantage for women on spatial test performance calls for more training of these skills. On the other hand, such disadvantage at the advanced level calls into question its importance for future success. More research on domain-specific spatial abilities in architecture, particularly among experts, is therefore needed. Finally, three new spatial ability tests with sufficient difficulty (i.e., no ceiling effects) are available for further research, development and application in the context of architecture as well as in other domains. Since the tests do not require prior knowledge and share common processes with existing tests, they are suitable for use beyond architecture.

## Data Availability Statement

The raw data supporting the conclusions of this article along with syntax and test items are publically available at: https://osf.io/jf5mx/.

## Ethics Statement

The studies involving human participants were reviewed and approved by the Ethics Committee of ETH Zurich. The patients/participants provided their written informed consent to participate in this study.

## Author Contributions

All authors listed have made a substantial, direct and intellectual contribution to the work, and approved it for publication.

## Conflict of Interest

The authors declare that the research was conducted in the absence of any commercial or financial relationships that could be construed as a potential conflict of interest.

## Figures and Tables

**TABLE A1:** Infit and outfit measures per item.

**Scale**	**Item**	**Infit MSQ**	**Outfit MSQ**	**Mean**
ULT	A1_plan	0.921	0.809	0.89
	B1_plan	0.927	0.924	0.83
	A2_axo	0.962	0.974	0.81
	B2_axo	1.084	1.155	0.70
	A3_plan	1.011	0.996	0.61
	B3_plan	1.040	0.994	0.81
	A4_axo	0.916	0.824	0.85
	B4_axo	1.007	1.070	0.70
	A5_plan	1.071	1.096	0.65
	A6_axo	0.950	0.926	0.74
	B6_axo	0.973	0.928	0.61
	A7_plan	0.964	0.961	0.48
	B7_plan	0.991	1.028	0.63
	A8_axo	0.963	0.946	0.48
	B8_axo	0.987	0.986	0.68
Packing	Pack1.1	0.910	0.833	0.86
	Pack1.2	1.138	1.205	0.62
	Pack1.3	0.996	1.002	0.49
	Pack1.4	0.917	0.755	0.87
	Pack2.1	0.898	0.837	0.79
	Pack2.2	1.040	1.061	0.72
	Pack2.3	0.847	0.778	0.65
	Pack2.4	0.910	0.884	0.41
Indoors	Indr1	1.024	1.036	0.65
	Indr2	1.148	1.187	0.64
	Indr3	0.855	0.831	0.72
	Indr4	0.992	0.974	0.81
	Indr5	0.870	0.812	0.84
	Indr6	0.952	0.944	0.53
	Indr7	0.823	0.769	0.89
	Indr8	1.068	1.095	0.64
				

**TABLE A2:** Number of items by test version and respective reliability estimates.

**Measure**	**# items**			**Cronbach’s α (95% CI)**		**Ordinal Omega total (95% CI)**	
			
	**Study 1**	**Study 2**	**Overlap**	**Study 1**	**Study 2**	**Study 1**	**Study 2**
ULT	8	10	6	0.57 (0.52–0.62)	0.55 (0.45–0.65)	0.72 (0.68–0.75)	0.64 (0.56–0.72)
Packing	8	10	7	0.53 (0.47–0.58)	0.46 (0.33–0.58)	0.69 (0.66–0.73)	0.61 (0.53–0.70)
IPT	8	10	5	0.44 (0.37–0.51)	0.66 (0.58–0.73)	0.64 (0.60–0.69)	0.78 (0.73–0.83)
MCT	10	10	10	0.70 (0.67–0.74)	0.62 (0.54–0.71)	0.84 (0.82–0.86)	0.78 (0.73–0.83)
MRT	24	24	24	0.86 (0.85–0.88)	0.84 (0.81–0.88)	0.93 (0.92–0.94)	0.92 (0.90–0.94)
							

**TABLE A3:** Descriptive statistics of test performance in study 2 (*N* = 117) for the full test versions.

	**Raw Mean (*SD*)**	**Mean proportion correct (*SD*)**	**Skew**	**Min**	**Max**	**N Items**
ULT	15.07 (2.94)	0.75 (0.15)	−0.43	7	20	10
IPT	7.14 (2.26)	0.71 (0.23)	−0.73	1	10	10
Packing	6.85 (1.81)	0.69 (0.18)	−0.37	2	10	10
						

## Footnotes

1 We use the term ‘gender’ rather than ‘sex’ because it is associated with a broader meaning (i.e., both birth-assigned sex and gender identity). However, we use the terms ‘males’ and ‘females’ rather than ‘men’ and ‘women’ because these are the labels with which participants were asked to indicate their gender, without reference to birth-assigned sex or gender identity.

2 In a preliminary analysis, we also included institute as an independent variable, because gender distributions differed between schools. The results were highly similar with respect to gender [*F*(6,573) = 11.58, *p* < 0.001, η_*p*_^2^ = 0.11] and degree [*F*(6,573) = 5.83, *p* < 0.001, η_*p*_^2^ = 0.06]. The main effect of institute and the interaction effects did not reach significance. Therefore, for simplicity, we report the analysis across institutes.

3 This distinction may seem artificial because the array of ‘multiple objects’ in ULT can be considered one object, and the features of the ‘single’ object in IPT as multiple objects. However, considering these stimuli are designed to simulate real life objects, the distinction seems relevant (i.e., we consider a view of streets and buildings as multiple objects, and single buildings as single objects).
